# Plasma Hormones Facilitated the Hypermotility of the Colon in a Chronic Stress Rat Model

**DOI:** 10.1371/journal.pone.0031774

**Published:** 2012-02-20

**Authors:** Chengbai Liang, Hesheng Luo, Ying Liu, Jiwang Cao, Hong Xia

**Affiliations:** Department of Gastroenterology, Renmin Hospital of Wuhan University, Wuhan, China; Kaohsiung Chang Gung Memorial Hospital, Taiwan

## Abstract

**Objective:**

To study the relationship between brain-gut peptides, gastrointestinal hormones and altered motility in a rat model of repetitive water avoidance stress (WAS), which mimics the irritable bowel syndrome (IBS).

**Methods:**

Male Wistar rats were submitted daily to 1-h of water avoidance stress (WAS) or sham WAS (SWAS) for 10 consecutive days. Plasma hormones were determined using Enzyme Immunoassay Kits. Proximal colonic smooth muscle (PCSM) contractions were studied in an organ bath system. PCSM cells were isolated by enzymatic digestion and *IKv* and *IBKca* were recorded by the patch-clamp technique.

**Results:**

The number of fecal pellets during 1 h of acute restraint stress and the plasma hormones levels of substance P (SP), thyrotropin-releasing hormone (TRH), motilin (MTL), and cholecystokinin (CCK) in WAS rats were significantly increased compared with SWAS rats, whereas vasoactive intestinal peptide (VIP), calcitonin gene-related peptide (CGRP) and corticotropin releasing hormone (CRH) in WAS rats were not significantly changed and peptide YY (PYY) in WAS rats was significantly decreased. Likewise, the amplitudes of spontaneous contractions of PCSM in WAS rats were significantly increased comparing with SWAS rats. The plasma of WAS rats (100 µl) decreased the amplitude of spontaneous contractions of controls. The IKv and IBKCa of PCSMs were significantly decreased in WAS rats compared with SWAS rats and the plasma of WAS rats (100 µl) increased the amplitude of IKv and IBKCa in normal rats.

**Conclusion:**

These results suggest that WAS leads to changes of plasma hormones levels and to disordered myogenic colonic motility in the short term, but that the colon rapidly establishes a new equilibrium to maintain the normal baseline functioning.

## Introduction

The irritable bowel syndrome (IBS) is defined as a chronic and recurrent functional bowel disorder characterized by chronic abdominal pain, discomfort, bloating, and altered bowel habits in the absence of any detectable structural abnormalities or infection on routine testing [1], [2]. Altered visceral perception and motility are the main pathophysiologic factors of IBS [3]–[6].

Many studies have shown that IBS patients have various gastrointestinal (GI) motor disturbances that often arise from an exaggerated physiological response to stimuli such as diet and stress [7], [8]. The stress changes their bowel patterns and produces abdominal pain [9]. There is now compelling evidence for the modulatory role of physical and psychological stresses in the digestive disorders, whether acute or chronic [7]. In particular, epidemiological, empirical and clinical observations provide valuable support for life stress as a common co-morbid event in IBS, which may strongly influence symptom onset, severity, and persistence in certain IBS subtypes [10], [11]. The importance of psychological factors was underscored by a recent study that demonstrated significant relief with an open-label placebo given with the strong suggestion that placebos “produce significant mind-body self-healing processes.” [12]. Furthermore, IBS patients tend to be more vulnerable to stressful events in daily life [13]. They may also have an increased incidence of other functional, somatoform disorders, as demonstrated by an increased incidence of “prostatitis syndrome” (PS) or IBS in patients who have one or the other disorder [14].

It has been shown that stress causes changes in the secretion of brain-gut peptides and gastrointestinal hormones such as corticotropin releasing hormone (CRH), thyrotropin (TSH), motilin (MTL), vasoactive intestinal peptide (VIP), and so on [15], [16]. Brain-gut peptides are produced by gut endocrine cells, which are derived from neuroectoderm and can be grouped according to common cytochemical characteristics, such as the concept of the acronym APUD (amine precursor uptake and decarboxylation) [15]. Likewise, the gastrointestinal hormones constitute a group of hormones secreted by enteroendocrine cells in the stomach, pancreas, and small intestine that control various functions of the digestive organs. Enteroendocrine cells do not form endocrine glands but are scattered throughout the digestive tract. They exert autocrine and paracrine actions that integrate some of the gastrointestinal functions [17]. These endogenous peptides and hormones play a role in mediating stress-induced changes in gastrointestinal motor function [18], [19] and colonic permeability [20], [21] as well as rectal sensitivity [22], [23]. Furthermore, evidence in patients with IBS suggests that some peptides and/or hormones play a significant role in mediating stress-induced alterations of gastrointestinal motor function [10], [24], [25].

For the terminal effect of these peptides and hormones on motor function, permeability and sensitivity, either directly or indirectly, ion channels play an important role, such as in controlling the manner and extent of colonic motility [26]. The main types of K^+^ channels associated with motility of the colon are the Kv and BKca channels, which include trans-membrane proteins that form the tunnels for the ions and regulatory sub-units. Peptides and hormones affect these sub-units directly or indirectly to control the K^+^ currents, so as to govern the motility of the circular smooth muscle (CM) and longitudinal smooth muscle (LM). The strength of contractions generated by LM and CM layers are largely determined by the amplitude, duration and frequency of action potentials, which mediate the rapid influx of Ca^2+^ into smooth muscle cells and the subsequent activation of the contractile machinery. Potassium (K^+^) channels actively participate in shaping the electrical activity of smooth muscle to generate outward currents [27]. The opening of K^+^ channels is associated with restoration of the resting potential and inhibition of contractile activity. The diversity in the types of K^+^ channels found in smooth muscle of the gut reflects the capacity of this system to fine tune the electrical activity of the syncytium to control intestinal rhythm. This syncytium is formed by smooth muscle cells connected to each other both anatomically and electrically through gap junctions which allow current to flow intercellularly from one cell to the other, thus enabling the mechanical activity of the constituent smooth muscle cells to be coordinated [27]. The high conductances of BK channels provide ideal negative feedback regulators in many cell types by decreasing voltage-dependent Ca^2+^ entry through membrane potential hyperpolarization. These channels were first studied in smooth muscle cells where they are the key players in setting contractile tone [28].

However, the mechanism through which stress contributes to colonic motility in IBS patients remains elusive. The repetitive water avoidance stress (WAS) model stimulates the desire to survive as well as fear of the surrounding environment. It imposes the stress of being forced to adapt, mimics the pattern of daily stress experienced by humans, and is a well-accepted animal model for studying the possible mechanisms involved in altered colonic motility [16].

Therefore, our hypothesis was that compared with controls, WAS rats would have some changes of fecal output in different environments and alterations in the secretion of peptides and hormones and in the function of the smooth muscle of the colon, aside from any pathological changes.

## Materials and Methods

### Animals

Adult male Wistar rats (200–250 g) were purchased from the disease control center of Hubei Province. Animals were maintained on a normal light-dark cycle, housed in pairs, and provided with food and water ad libitum. All protocols were approved by the Institutional Animal Care and Use Committee of Wuhan University (Approval ID: WHU20110312) and adhered to the ethical guidelines of the International Association for the Study of Pain.

### Water avoidance stress protocol

As previously described [29], rats were placed on a block (10×8×8 cm) affixed to the center of a plexiglas cage (45×25×25 cm), filled with fresh room temperature water (25°C) to within 1 cm of the top of the block (WAS) or kept empty (sham WAS), for 1 h daily, for 10 consecutive days.

### Measurement of fecal pellet output and rat weight

We used a validated procedure to estimate autonomic regulation of distal colonic motility during the WAS [30], [31]. Fecal pellets found in the tank were counted at the end of each 1-h WAS or sham WAS session. The weight of the rats was measured every day before exposure to WAS or sham WAS to evaluate weight change from baseline. To measure the baseline colonic motility after the 10-day testing period, we put the WAS or sham WAS rats in stand-alone cages for 1 hour on Day 11, after which the fecal pellets were counted. Subsequently, the colonic motility of the rats was studied using the acute stress model as described previously [32]. All rats were lightly anesthetized with ethyl-ether on Day 11. The fore shoulders, upper forelimbs, and thoracic trunk of rats were then wrapped in a confining harness of paper tape to restrict the body movements of rats, following which the rats were placed in their home cage for 1 h after which the fecal pellets were counted.

### ELISA assay

Rats were anesthetized with ether. The blood from the heart was collected in EDTA (ethylenediamine tetra-acetic acid) tubes and spun for 10 min at 3000 rpm. Plasma was pipetted out and stored under −80°C for further gastrointestinal hormone analysis. Brain-gut peptides and gastrointestinal hormones were determined using an Enzyme Immunoassay Kit (R&D, Inc., MI, U.S.A) at a dose of 10 µL plasma per sample per well for the assay according to the manufacturer's instructions. Samples were analyzed in duplicate in a single assay.

### Preparation of isolated colonic smooth muscle strips

Rats were anesthetized with ether, followed by sacrifice along the ventral midline. The proximal colon was removed, cleaned and opened along the mesenteric border and then placed in Ca^2+^-free physiological saline solution (PSS) (in mol/L): NaCl 147.0, KCl 4.0, CaCl_2_ 2.0, NaH_2_PO_4_ 0.42, Na_2_HPO_4_ 2.0, MgCl_2_ 1.05, and glucose 5.5 (adjusted to pH 7.4 with NaOH) bubbled with carbogen (95% O_2_/5% CO_2_). The digestive solution contained 0.1% (w/v) collagenase II, 0.1% (w/v) trypsin inhibitor and 0.2% (w/v) bovine serum albumin (BSA) dissolved in Ca^2+^-free PSS. The internal pipette solution used in experiments investigating APs and RPs contained (in mmol/L): K-aspartate 120.0, HEPES 10.0, EGTA 10.0, Na_2_ATP 5.0, MgCl_2_ 4.0, and CaCl_2_ 3.0 (adjusted to pH 7.4 with KOH). The internal pipette solution of the large conductance potassium channel contained (in mmol/L): KCl 20.0, K-aspartate 110.0, MgCl_2_·6H2O 1.0, Mg-ATP 5.0, Na_2_ creatine phosphate 2.5, EGTA 2.5, and 2.0 µg/mL nystatin (adjusted to pH 7.4 with KOH). The smooth muscle strips (3 mm×7 mm) were obtained after the mucosa and submucosa were excised. When studying longitudinal smooth muscles (LM), the strips parallel to the mesenteric were used and when studying circular smooth muscles (CM), the strips vertical to the mesenteric were used.

### Preparation of isolated colonic smooth muscle cells (SMCs)

Single SMCs were isolated by enzymatic digestion [27]. The strips of proximal colon were pinned to the base of the Sylgard surface of a Petri dish and the mucosa and submucosa was carefully dissected away under an anatomical microscope. The tissue was cut into small strips (about 2 mm×5–6 mm) and placed in Ca^2+^-free PSS solution that contained 0.12% (w/v) collagenase II supplemented with 0.2% soybean trypsin inhibitor and 0.2% BSA, and incubated for 19–34 min at 37°C. After completion of digestion, the segments were washed five times in a Ca^2+^-free PSS solution and then triturated gently with a fire-polished Pasteur pipette to create a cell suspension. Cells were stored at 0–4°C and used within 8 h.

### Contraction recording of proximal colonic smooth muscle (PCSM) strips

Each fresh smooth muscle strip was mounted in an organ bath and connected to an isometric force transducer (JZJOIH, Chengdu, China). The organ baths contained 6 mL Tyrode's buffer at 37°C and were constantly warmed by a circulating water jacket at 37°C and bubbled with carbogen (95% O_2_/5% CO_2_). One end of the strip was fixed to a hook on the bottom of the chamber, while the other end was connected by a thread to an external isometric force transducer at the top. Each muscle strip was placed under a resting preload of 1.0 g to obtain a maximum response to 1 M ACh and allowed to equilibrate for 60 min. During the equilibration period, the sections were washed every 20 min with Tyrode's and the basal tension was maintained. To obtain a stable and acceptable level of sensitivity before the experimental procedure began, the colonic section was challenged with 40 mM KCl until reproducible responses were obtained. The frequencies of contraction were calculated by counting the contraction waves per 20 minutes. The mean contractile amplitude and frequency of spontaneous contractions were recorded (WAS and sham WAS values) and compared with the mean contractile amplitude and frequency when the control strips were exposed to 100 µl WAS plasma or sham WAS plasma. Heparin was used as the anti-coagulant in the plasma instead to avoid possible influence of the electrolytes caused by EDTA. The results are presented as the changed percentage (changed percentage = 100%×(response value – control value)/control value).

### Whole-cell patch-clamp recordings

Several drops of cell suspensions were placed in a recording chamber that was mounted with an inverted microscope (Olympus, Japan). After adhering to the coverslip, the cells were infused with Tyrode's buffer (3 mL/min). Pipettes were made using a micropipette puller (P-97; Sutter, USA). Typical pipette resistances were 3–5 MΩ. A gigaseal was formed with negative suction. Capacitance was compensated for and the residual capacitance current was removed digitally. Whole-cell currents were recorded with an EPC-10 amplifier (HEKA, Germany). Acquisition and analysis of data were accomplished by using PulseFit (HEKA Instrument, Germany). The internal (pipette) solution for recording IK (V) contained (in mM) 125 KCl, 4 MgCl_2_, 10 HEPES, 10 EGTA, and 5 Na_2_ATP (pH 7.3).The values of WAS and sham WAS IBKca and IKv currents were recorded and the effects of WAS plasma or sham WAS plasma at different concentrations were investigated on IBKca and IKv when added to control rats. Data were filtered at 200 Hz, digitized at 10 kHz (filter 1) and 2.9 kHz (filter 2), and stored in the computer for subsequent analyses. All the experiments were conducted at 25±2°C.

### Statistical analysis

All the analyses were carried out using SPSS 13.0 (SPSS Inc., Chicago, IL). Data are expressed as mean ± S.E.M. “n” is the number of cell analyses isolated from at least five animals. ANOVA with post hoc Bonferroni's correction was used for the comparisons of more than two groups, and Student's t-test was used for the comparison of paired samples. P<0.05 was considered statistically significant.

## Results

### Comparison of fecal pellet output in WAS rats and SWAS rats

**Figure 1:**
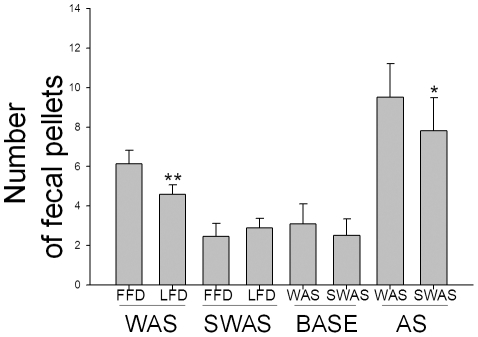
Number of fecal pellets. Number of the fecal pellets in water avoidance stress (WAS) and sham water avoidance stress (SWAS) rats at the first five days (FFD) or the last five days (LFD) and at the base state (BASE) or acute stress state (AS) on the eleventh day. Data are expressed as mean ± SEM (n = 10). Statistical significance of difference between fecal pellet counts in WAS and SWAS rats at FFD or LFD and BASE or AS was evaluated by using unpaired Student's t-test. **P<0.01, * P<0.05 compared between WAS and SWAS.

The fecal pellet output in all animals was significantly increased by WAS with exposure to the water for 1 hour, and was more for the first five days (FFD) than for the last five days (LFD) (6.14±0.69 vs. 4.88±0.50, p<0.01, Figure 1). However, there was no such effect with exposure to sham stress (2.46±0.67 vs. 2.42±0.23, p>0.05, Figure 1). The basal fecal pellet output of the WAS rats at day eleven showed no significant difference from that of the SWAS rats (3.1±0.99 vs. 2.5±0.84, p = 0.164, Figure 1). When exposed to the acute stress situation, the WAS rats had a greater fecal pellet output than the SWAS rats (9.5±1.72 vs. 7.8±1.68, p<0.05, Figure 1).

### Plasma Concentrations of brain-gut peptides

We analyzed the plasma brain-gut peptides and gastrointestinal hormone levels in the WAS and SWAS rats. As Table 1 shows, the concentrations of SP, TRH, CCK and MTL were significantly increased in the WAS rats compared to those in the SWAS rats, while the concentrations of VIP, CGRP and CRH showed no significant difference between the two groups. Furthermore, the plasma levels of PPY in WAS rats were significantly lower than those in SWAS rats.

**Table 1:** Plasma brain-gut peptides and gastrointestinal hormones in WAS and SWAS.

Peptide	SWAS	WAS
SP	58.26±7.97 ng/L	92.44±15.76 ng/L*
VIP	52.72±3.41 ng/L	48.213±4.13 ng/L
TRH	1.02±0.25 ulU/ml	2.12±0.16 ulU/ml*
CGRP	22.96±3.48 ng/L	25.09±4.94 ng/L
CRH	18.86±0.97 ng/L	19.29±0.88 ng/L
MTL	66.53±7.49 pg/L	111.69±2.41 pg/L**
CCK	75.51±19.23 ng/L	153.05±25.41 ng/L**
PYY	85.48±20.53 ng/L	43.67±12.03 ng/L**
* P<0.05	**P<0.01	

### WAS increased spontaneous contractile activity of proximal colonic smooth muscle strips in vitro

**Figure 2:**
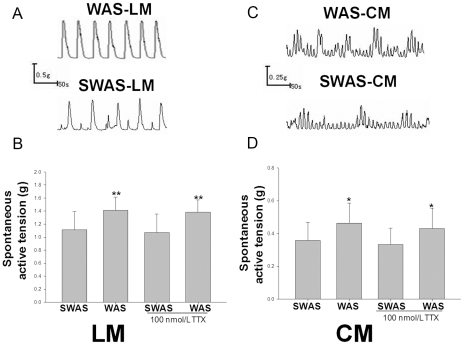
WAS increased spontaneous contractile amplitude of proximal colonic smooth muscle (PCSM) strips. A, C: Representative traces of the effect of WAS or SWAS on the spontaneous contractile amplitude of longitudinal smooth muscle (LM) and circular smooth muscle (CM) strips. WAS increased the mean contractile amplitude of CM and LM strips in the absence and presence of 100 nm TTX. B, D: Summarized results of the LM (B) and CM (D) spontaneous contractions in WAS and SWAS rats. Data are expressed as mean ± SEM (n = 12). Significant difference is indicated by * (P<0.05 compared to SWAS or SWAS-TTX groups, respectively, according to the unpaired Student's t-test).

The baseline amplitude of spontaneous contractile activities from the WAS rat colons was significantly increased when compared to that of the SWAS rats (Figure 2A, 2C). The mean active tension of longitudinal smooth muscle (LM) in WAS rats was significantly higher than that in SWAS rats (1.41±0.2 g vs. 1.12±0.27 g, P<0.01, Figure 2A, 2B) and the mean active tension of circular smooth muscle (CM) in WAS rats was significantly higher than that in SWAS rats (0.46±0.11 g vs. 0.35±0.07 g, P<0.05, Figure 2C, 2D). However, the frequency of spontaneous contractions of CM or LM from WAS rats did not change significantly (data not shown) when compared to that of SWAS rats. When tetrodotoxin (TTX, 100 nM) was added into the organ bath for 30 min to block the influence of the neuronal factors in the enteric nervous system on smooth muscle contraction, the spontaneous contractions decreased in the two groups, but the difference between them still persisted. The frequency of spontaneous muscle strip contractions was again not significantly different between the two groups.

### WAS plasma decreased spontaneous contractile amplitude of proximal colonic smooth muscle (PCSM) strips

**Figure 3:**
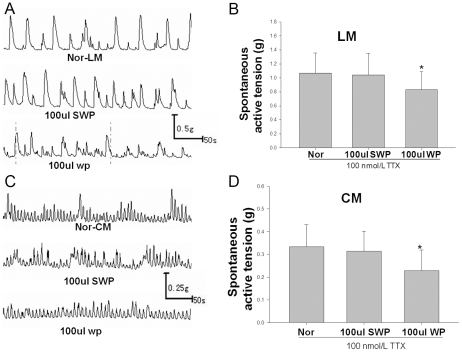
WAS plasma decreased spontaneous contractile amplitude of proximal colonic smooth muscle (PCSM) strips. A, C: Representative traces of the effect of WAS rat plasma (WP) or SWAS rat plasma (SWP) on the spontaneous contractile amplitude of longitudinal smooth muscle (LM) and circular smooth muscle (CM) strips. WP decreased the mean contractile amplitude of CM and LM strips in the presence of 100 nm TTX. B, D: Summarized results showing the effect of WP or SWP on LM (B) and CM (D) spontaneous contractions in normal rats. Data are expressed as mean ± SEM (n = 8). Significant difference is indicated by * (P<0.05 compared to normal (Nor) or Nor-SWP group according to the ANOVA analysis).

The amplitudes of contractile activities of LM and CM with added WAS rat plasma (WP 100 ul/6 ml) were significantly decreased (1.07±0.29 g vs. 0.83±0.31 g and 0.31±0.1 g vs. 0.23±0.82 g, respectively, p<0.05, Figure 3A–D) in the presence of 100 nM TTX in the organ bath. However, the amplitude of contractile activities with added SWAS rat plasma (SWP 100 ul/6 ml) was not significantly changed (p>0.05). The frequency of spontaneous contraction activities of CM or LM from WAS rats was not significantly changed in the absence and presence of WP or SWP (data not shown here).

### Effect of WAS on *IKV* and *IBKca* current in PCSMCs

**Figure 4:**
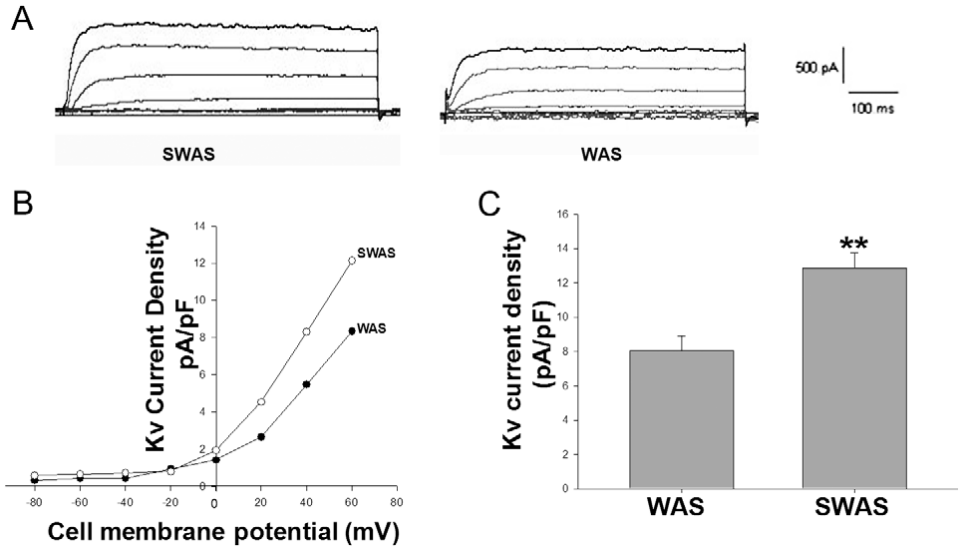
WAS reduced the whole-cell *IKv* of PCSMC. A: Current tracings elicited by +20 mV depolarizing steps from a constant holding potential of −80 mV to +60 mv; the basal *IKv* was reduced in PCSMCs from WAS rats compared with SWAS rats. B: Current-voltage relationships comparing *IKv* current density between PCSMCs from WAS and SWAS rats. C: Summarized data showing the density of the currents at 60 mV in WAS and SWAS PCSMCs. **: P<0.01 vs. SWAS PCSMCs.

**Figure 5:**
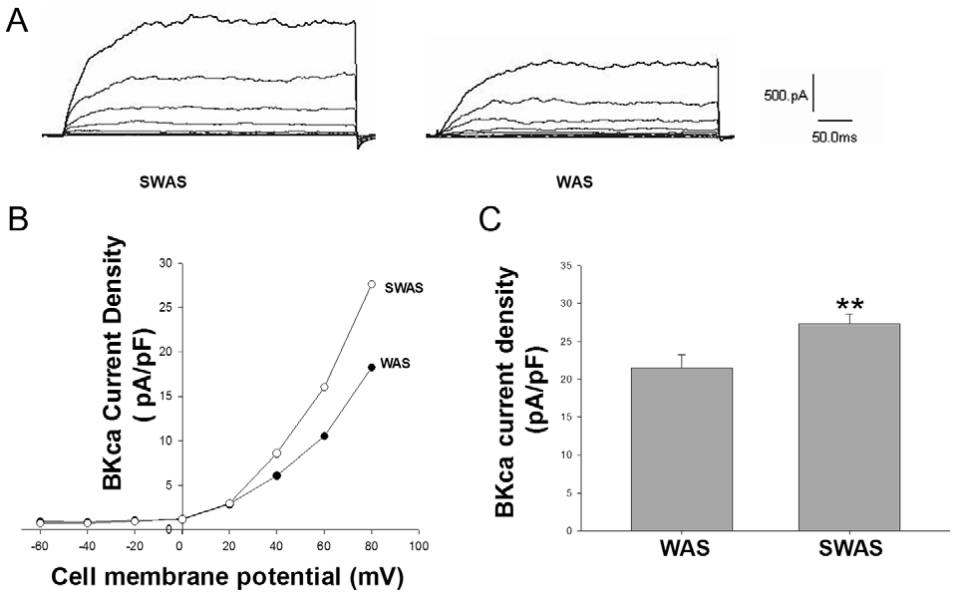
WAS reduced the whole-cell *IBKca* of PCSMC. A: Current tracings elicited by +20 mV depolarizing steps from a constant holding potential of −60 mV to +80 mv; the basal *IBKca* current was reduced in PCSMCs from WAS rats compared with SWAS rats. B: Current-voltage relationships comparing *IBKca* density between PCSMCs from WAS and SWAS rats. C: Summarized data showing the density of the currents at 80 mV in WAS and SWAS PCSMCs. **: P<0.01 vs. SWAS PCSMCs.

With whole-cell voltage-clamp recordings, a delayed rectifier *I*K was found to be the predominant Kv current in freshly isolated rat PCSMCs (n = 28/30). *I*K was activated slowly at membrane potentials from positive to −20 mV with no inactivating kinetics over the stimulation period (600 ms). The transient and fast-inactivating *I*A was rare in these cells (2/30). As shown in Figure 4, the IKv of WAS rats is decreased when compared to that of SWAS rats (8.05±0.86 pA/pF vs. 12.86±0.91 pA/pF, p<0.01) at +60 mv). The large conductance IBKCa current was detected by using a depolarizing step pulse from a holding potential of −60 mV to +80 mV for 400 ms (Δv = +20 mv). As shown in Figure 5, the IBKca current density of WAS rats was decreased when compared to that of SWAS rats (21.49±1.75 pA/pF vs. 27.20±1.30 pA/pF, p<0.01) at +80 mv.

### Effect of WAS rat plasma on IKV and IBKca current in rat PCSMCs

**Figure 6:**
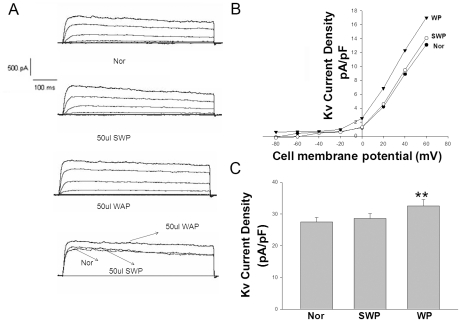
Excitation of *I*Kv in rat PCSMCs by WAS rat plasma (WP). *A*: Representative record of *I*K in the absence and presence of WP or SWP. B: I–V relationships of IK in the absence and presence of WP or SWP. C: Summarized data showing the density of the currents at 80 mv in WAS and SWAS PCSMCs. **: P<0.01 vs. SWAS PCSMCs according to the ANOVA analysis.

**Figure 7:**
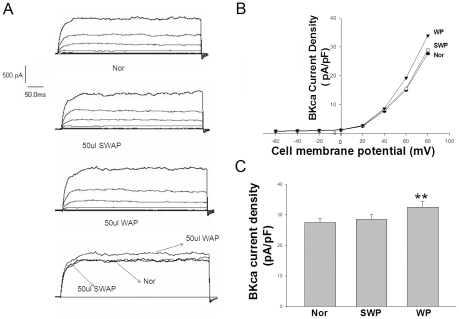
Excitation of *IBKca* in rat PCSMCs by WAS rat plasma. *A*: Representative records of *I*K in the absence and presence of WP or SWP (50 ul/2 ml). B: I–V relationships of IBKca in the absence and presence of WP OR SWP. C: Summarized data showing the density of the currents at 80 mV in WAS and SWAS PCSMCs. **: P<0.01 vs. SWAS PCSMCs according to the ANOVA analysis.

To explore the effect of plasma on IKV and IBKca current in proximal colon SMCs, we took the plasma of the WAS or SWAS rats to perfuse the PCSMCs of normal Wistar rats. As shown in Figure 6, the plasma of the WAS or SWAS rats activated the IKv current in freshly isolated normal rat PCSMCs (from 13.05±0.66 pA/pF to 17.10±1.27 pA/pF, p<0.01, or to 13.69±0.78 pA/pF, respectively) at +60 mv. As shown in Figure 7, the plasma of the WAS or SWAS rats also activated BKCa current in freshly isolated control rat proximal colon SMCs (from 27.55±1.33 pA/pF to 32.54±2.02 pA/pF, p<0.01, or to 28.59±1.49 pA/pF, respectively).

## Discussion

The WAS rat model is regarded as an acceptable and classical model of IBS [26], and it has been used to investigate mechanisms of colonic motility [15]. Yet, the definitive pathogenesis of colonic motility associated with WAS is not available [26], [33], [34]. The present study showed the differences of amplitude and frequency of spontaneous contraction of proximal colonic strips between WAS rats and SWAS rat, and the changes of amplitude of IKv and IBKca currents in proximal colon SMCs in the two groups. These data support our hypothesis that WAS induces disordered colonic motility, which is associated with the dys-synchrony of brain-gut peptides and gastrointestinal hormones and with enhanced myogenic contraction of the proximal colon.

The WAS decreased the transit times of the proximal colon. Our results show that the WAS treatment resulted in a significantly higher number of fecal pellets during the 1 h acute restraint stress treatment in the WAS rats compared to the SWAS rats, indicating that WAS rats were over-reactive to the acute stress. This is consistent with the studies of Mayer EA and Welgan P [29], [35]. Curiously, our results also showed that there was no significant difference of fecal pellet numbers in WAS rats in the resting state on Day 11 compared with SWAS rats. Also, the WAS rats adapted to their chronic stress based on the fecal pellet numbers and behavioral responses in the first five days compared to the last five days. These results are consistent with the clinical observation that IBS patients aren't intolerant of daily stressful events but develop irritable or spastic colon under various acute and more extreme stressors. We presume that the normal fecal pellet counts in WAS rats on Day 11 after 10 days of chronic stress occurred because of adaptive functioning, as the brain-gut axis, autonomic nervous system, gastrointestinal hormones and colon SMCs came to a new homeostasis. This could be due to central nervous system reintegration around the chronic stressor, such as by changing the expression of some proteins [2], [36] or by the association of synapses to guide chronic stress hormone secretion. This would balance microscopic but critical changes of colon smooth muscle induced by long-term chronic stress, as the PCSM is in a dynamic equilibrium and could adapt to mild and non-ulcerogenic new stresses. Accordingly, we studied representative brain-gut peptides and gastrointestinal hormones as well as the motility of proximal colon to explore restrictive relationships between them.

In our present study, the WAS rats had higher levels of SP, TRH, MTL and CCK in plasma and lower levels of PYY when compared with SWAS rats. The effects of each hormone in IBS patients or animal models have been researched extensively with controversial results [37]–[40] focused upon relative or absolute secretory volume. Because each group or individual experiences different stressors as well as different cultures, genetic backgrounds, living habits and so on, they have responded differently to cope with stress [8]. We must regard secretion of brain-gut peptides and GI hormones as a whole biological activity of the organism. The results showed that the WAS rat plasma decreased the amplitude of proximal colon muscle strips in the presence of 100 nM TTX compared to controls (Figure 3) and increased the IKv and IBKca current density of proximal colon SMCs (Figures 4, 5, and 6), but that the plasma of SWAS rats didn't. This showed that the abnormal levels of brain-gut peptides and GI hormones in the plasma of WAS rats have been demonstrated in control rat preparations to correlate with slow colon transit. But the myogenic changes in the colons of the rats showed the opposite results (Figure 2), with the amplitude of spontaneous contractions increased in the presence of 100 nM TTX or not and with decreased IKv and IBKca current density after WAS treatment (Figures 4, 5, and 6), whereas the SWAS rats didn't have significant changes; in other words, chronic stress increased colonic motility in the WAS rats compared to the SWAS rats.

Brain-gut peptides and GI hormones affect colonic motility by two fundamentally different pathways: acting on neurons in the myenteric plexus and acting directly on the smooth muscle cells. These differential effects depend on the density of receptors for these peptides and hormones that exist in myenteric plexus and CM or LM [41]–[44] as well as the subtypes of receptors in the tissue where the activation occurs and on environmental factors [20]. These peptides and hormones interact with each other in vivo, in addition to their different affinities for the receptors, and also act via endocrine, paracrine and neurocrine pathways [45]. The brain-gut hormones and GI hormones are secreted into blood, and hence circulate systemically, where they affect function of other parts of the digestive tract, liver, pancreas, brain and a variety of other targets. Some of these peptides inhibits ghrelin secretion and exert opposite effects on hypothalamic neuronal activity and gastric emptying. The physiological actions of peptides or hormones involve coupling their respective receptors to G-proteins that stimulate cAMP/cGMP-mediated signaling cascades [46]. To summarize, this body of research supports our findings that gastrointestinal hormones and the brain-gut peptides can directly interact with their associated receptors in smooth muscle cells, thus influencing muscle tone.

This study has the following limitations: First, we have not tested whether the changed GI motility is associated with changes in the nervous system including the myenteric nerve plexus, submucosal plexus and rhythm of the interstitial cells of Cajal (ICC) in addition to the association with peptides and hormones. This deserves further investigation. Second, we have not studied the potential links between stress and non-stress induced peptides and hormones. The reasons that female rats were not selected for this study was to avoid potential effects of estrogen in motility modulation.

In summary, we found increased motility of proximal colon smooth muscle in WAS rats, that plasma of WAS rats inhibited the contraction of proximal colon in control rats, and that WAS rats had the same fecal pellet count in the unstressed state but an increased count in the acute stress state compared with SWAS rats. We concluded that WAS rats adapted well in the normal state unless an acute stress event occurred. This prompts us to speculate that in IBS patients, there may be less need to control the motility of colon in the normal state and more need to focus on maintaining that state and avoiding or better managing the occurrence of acutely stressful events.

## References

[1] Lee OY (2010). Asian motility studies in irritable bowel syndrome.. J Neurogastroenterol Motil.

[2] Ren TH, Wu J, Yew D, Ziea E, Lao L (2007). Effects of neonatal maternal separation on neurochemical and sensory response to colonic distension in a rat model of irritable bowel syndrome.. Am J Physiol Gastrointest Liver Physiol.

[3] Ait-Belgnaoui A, Bradesi S, Fioramonti J, Theodorou V, Bueno L (2005). Acute stress-induced hypersensitivity to colonic distension depends upon increase in paracellular permeability: role of myosin light chain kinase.. Pain.

[4] Kellow JE, Eckersley CM, Jones MP (1991). Enhanced perception of physiological intestinal motility in the irritable bowel syndrome.. Gastroenterology.

[5] Bradette M, Delvaux M, Staumont G, Fioramonti J, Bueno L (1994). Evaluation of colonic sensory thresholds in IBS patients using a barostat. Definition of optimal conditions and comparison with healthy subjects.. Dig Dis Sci.

[6] Whitehead WE, Palsson OS (1998). Is rectal pain sensitivity a biological marker for irritable bowel syndrome: psychological influences on pain perception.. Gastroenterology.

[7] Maunder RG, Levenstein S (2008). The role of stress in the development and clinical course of inflammatory bowel disease: epidemiological evidence.. Curr Mol Med.

[8] Chrousos GP (2009). Stress and disorders of the stress system.. Nat Rev Endocrinol.

[9] Drossman DA, Sandler RS, McKee DC, Lovitz AJ (1982). Bowel patterns among subjects not seeking health care. Use of a questionnaire to identify a population with bowel dysfunction.. Gastroenterology.

[10] Murray CD, Flynn J, Ratcliffe L, Jacyna MR, Kamm MA (2004). Effect of acute physical and psychological stress on gut autonomic innervation in irritable bowel syndrome.. Gastroenterology.

[11] Bennett EJ, Tennant CC, Piesse C, Badcock CA, Kellow JE (1998). Level of chronic life stress predicts clinical outcome in irritable bowel syndrome.. Gut.

[12] Kaptchuk TJ, Friedlander E, Kelley JM, Sanchez MN, Kokkotou E (2010). Placebos without deception: a randomized controlled trial in irritable bowel syndrome.. PLoS One.

[13] Whitehead WE, Crowell MD, Robinson JC, Heller BR, Schuster MM (1992). Effects of stressful life events on bowel symptoms: subjects with irritable bowel syndrome compared with subjects without bowel dysfunction.. Gut.

[14] Vicari E, La Vignera S, Arcoria D, Condorelli R, Vicari LO (2011). High frequency of chronic bacterial and non-inflammatory prostatitis in infertile patients with prostatitis syndrome plus irritable bowel syndrome.. PLoS One.

[15] Welberg LA, Seckl JR (2001). Prenatal stress, glucocorticoids and the programming of the brain.. J Neuroendocrinol.

[16] Bradesi S, Schwetz I, Ennes HS, Lamy CM, Ohning G (2005). Repeated exposure to water avoidance stress in rats: a new model for sustained visceral hyperalgesia.. Am J Physiol Gastrointest Liver Physiol.

[17] Jaworek J, Nawrot-Porabka K, Leja-Szpak A, Konturek SJ (2010). Brain-gut axis in the modulation of pancreatic enzyme secretion.. J Physiol Pharmacol.

[18] Niederau C, Faber S, Karaus M (1992). Cholecystokinin's role in regulation of colonic motility in health and in irritable bowel syndrome.. Gastroenterology.

[19] Cassar-Malek I, Listral A, Picard B (1998). Contrôle hormonal des caractéristiques des fibres musculaires après la naissance.. Prod Anim.

[20] Larauche M, Kiank C, Tache Y (2009). Corticotropin releasing factor signaling in colon and ileum: regulation by stress and pathophysiological implications.. J Physiol Pharmacol.

[21] Sjolund K, Fasth S, Ekman R, Hulten L, Jiborn H (1997). Neuropeptides in idiopathic chronic constipation (slow transit constipation).. Neurogastroenterol Motil.

[22] Sabate JM, Gorbatchef C, Flourie B, Jian R, Coffin B (2002). Cholecystokinin octapeptide increases rectal sensitivity to pain in healthy subjects.. Neurogastroenterol Motil.

[23] Kamerling IM, Burggraaf J, van Haarst AD, Oppenhuizen-Duinker MF, Schoemaker HC (2003). The effect of motilin on the rectum in healthy volunteers.. Br J Clin Pharmacol.

[24] Posserud I, Agerforz P, Ekman R, Bjornsson ES, Abrahamsson H (2004). Altered visceral perceptual and neuroendocrine response in patients with irritable bowel syndrome during mental stress.. Gut.

[25] Monnikes H, Tebbe JJ, Hildebrandt M, Arck P, Osmanoglou E (2001). Evidence for stress-induced alterations in gastrointestinal motility and sensitivity.. Dig Dis.

[26] Zhang M, Leung FP, Huang Y, Bian ZX (2010). Increased colonic motility in a rat model of irritable bowel syndrome is associated with up-regulation of L-type calcium channels in colonic smooth muscle cells.. Neurogastroenterol Motil.

[27] Xu L, Yu BP, Chen JG, Luo HS (2007). Mechanisms mediating serotonin-induced contraction of colonic myocytes.. Clin Exp Pharmacol Physiol.

[28] Vogalis F (2000). Potassium channels in gastrointestinal smooth muscle.. J Auton Pharmacol.

[29] Mayer EA, Naliboff BD, Chang L, Coutinho SV (2001). V. Stress and irritable bowel syndrome. American journal of physiology.. Gastrointestinal and liver physiology.

[30] Vassallo M, Camilleri M, Phillips SF, Brown ML, Chapman NJ (1992). Transit through the proximal colon influences stool weight in the irritable bowel syndrome.. Gastroenterology.

[31] Venkova K, Johnson AC, Myers B, Greenwood-Van Meerveld B (2010). Exposure of the amygdala to elevated levels of corticosterone alters colonic motility in response to acute psychological stress.. Neuropharmacology.

[32] Gue M, Del Rio-Lacheze C, Eutamene H, Theodorou V, Fioramonti J (1997). Stress-induced visceral hypersensitivity to rectal distension in rats: role of CRF and mast cells.. Neurogastroenterology and motility: the official journal of the European Gastrointestinal Motility Society.

[33] Zou N, Lv H, Li J, Yang N, Xue H (2008). Changes in brain G proteins and colonic sympathetic neural signaling in chronic-acute combined stress rat model of irritable bowel syndrome (IBS).. Transl Res.

[34] Rojas-Macias V, Rodriguez-Fandino O, Jimenez-Ponce F, Saldivar-Gonzalez JA, Melendro-Lozano E (2010). External validity of a relevant model for irritable bowel syndrome (IBS) using chronic stress by water avoidance in Wistar rats.. Rev Gastroenterol Mex.

[35] Welgan P, Meshkinpour H, Hoehler F (1985). The effect of stress on colon motor and electrical activity in irritable bowel syndrome.. Psychosom Med.

[36] Bradesi S, Lao L, McLean PG, Winchester WJ, Lee K (2007). Dual role of 5-HT3 receptors in a rat model of delayed stress-induced visceral hyperalgesia.. Pain.

[37] Plotsky PM, Meaney MJ (1993). Early, postnatal experience alters hypothalamic corticotropin-releasing factor (CRF) mRNA, median eminence CRF content and stress-induced release in adult rats.. Brain Res Mol Brain Res.

[38] Simren M, Abrahamsson H, Bjornsson ES (2001). An exaggerated sensory component of the gastrocolonic response in patients with irritable bowel syndrome.. Gut.

[39] Heim C, Newport DJ, Heit S, Graham YP, Wilcox M (2000). Pituitary-adrenal and autonomic responses to stress in women after sexual and physical abuse in childhood.. JAMA.

[40] Zhang ZB, Zhang XY, Den JL, Hu JL, Zhang HB (1992). Preliminary studies on substance P and vasoactive intestinal polypeptide in ulcerativecolitis, chronic colitis and irritable bowel syndrome.. Biomedical Research.

[41] Gourcerol G, Wu SV, Yuan PQ, Pham H, Miampamba M (2011). Activation of corticotropin-releasing factor receptor 2 mediates the colonic motor coping response to acute stress in rodents.. Gastroenterology.

[42] Yuan PQ, Wu SV, Wang L, Tache Y (2010). Corticotropin releasing factor in the rat colon: expression, localization and upregulation by endotoxin.. Peptides.

[43] Rettenbacher M, Reubi JC (2001). Localization and characterization of neuropeptide receptors in human colon.. Naunyn Schmiedebergs Arch Pharmacol.

[44] Gross KJ, Pothoulakis C (2007). Role of neuropeptides in inflammatory bowel disease.. Inflamm Bowel Dis.

[45] Van Der Veek PP, Biemond I, Masclee AA (2006). Proximal and distal gut hormone secretion in irritable bowel syndrome.. Scand J Gastroenterol.

[46] Hillhouse EW, Grammatopoulos DK (2006). The molecular mechanisms underlying the regulation of the biological activity of corticotropin-releasing hormone receptors: implications for physiology and pathophysiology.. Endocr Rev.

